# Antivirulence Properties of Kuraridin Against Methicillin-Resistant *Staphylococcus aureus* (MRSA)

**DOI:** 10.3390/biomedicines13030564

**Published:** 2025-02-24

**Authors:** Nilakshi Barua, Ben Chung Lap Chan, Clara Bik-San Lau, Ping-Chung Leung, Kwok Pui Fung, Margaret Ip

**Affiliations:** 1 Department of Microbiology, Faculty of Medicine, Prince of Wales Hospital, The Chinese University of Hong Kong, Shatin, N.T, Hong Kong 999077, China; 2 Institute of Chinese Medicine and State Key Laboratory of Research on Bioactivities and Clinical Applications of Medicinal Plants, The Chinese University of Hong Kong, Shatin, N.T, Hong Kong 999077, China; benchan99@cuhk.edu.hk (B.C.L.C.);; 3 School of Biomedical Sciences, Faculty of Medicine, The Chinese University of Hong Kong, Shatin, N.T, Hong Kong 999077, China

**Keywords:** methicillin-resistant *Staphylococcus aureus*, Kuraridin, *C. elegans*, Sortase A, antivirulence, antimicrobial

## Abstract

**Background:** Methicillin-resistant *Staphylococcus aureus* (MRSA) is a major human opportunistic pathogen that causes a wide range of infections. The vast arsenal of virulence factors expressed remains the biggest challenge in treating MRSA with conventional antibiotic therapy. **Methods:** We investigated the effects of Kuraridin at subinhibitory minimum inhibition concentrations (MICs) of 1/8, 1/16, and 1/32 (concentrations that did not inhibit bacterial growth) on adhesion to fibrinogen, adhesion, internalization into HaCaT cells, and biofilm production in three MRSA strains representing the clonal types USA300, ST30, and ST239. **Results:** All three MRSA strains exhibited a significant decrease (*p* < 0.001) in adhesion to fibrinogen upon treatment with 1/8 and 1/16 MICs of Kuraridin. The adhesion and internalization of all the MRSA strains to HaCaT cells were decreased significantly (*p* < 0.001) upon treatment with the three subinhibitory concentrations of Kuraridin. The biofilm formation of USA300 (*p* < 0.001), ST30 (*p* < 0.001), and ST239 (*p* < 0.01) was significantly reduced at a 1/8 MIC. A significant decrease in biofilm formation at a 1/16 MIC was observed for USA300 (*p* < 0.001) and ST30 (*p* < 0.05). Confocal laser scanning microscopy (CSLM) analysis of the biofilms revealed a reduction in biofilm formation in the MRSA strain when treated with Kuraridin. In the in vivo *Caenorhabditis elegans* model, Kuraridin offered a sizable degree of protection against MRSA infection without being toxic to the nematode. **Conclusions:** Our findings reveal that Kuraridin has the potential to be an alternative antivirulence option for reducing MRSA pathogenicity.

## 1. Introduction

Methicillin-resistant *Staphylococcus aureus* (MRSA) has become a growing threat, causing outbreaks in hospitals and communities worldwide [1,2]. MRSA is frequently linked to bacteremia, skin and soft tissue infections, upper and lower respiratory tract infections, osteomyelitis, and septic arthritis. The pathogenicity of MRSA is controlled by a complex set of virulence factors, including exotoxins, enterotoxins, and bacterial biofilm formation [3]. Most MRSA virulence factors are covalently anchored to the peptidoglycan by cysteine transpeptidases known as sortase enzymes [4]. The enzyme Sortase A (SrtA) plays an essential role in the functional assembly of virulence factors associated with the adherence and invasion of the host tissue vital for systemic or localized infections [5]. MRSA infections pose a serious threat to life due to a lack of effective treatments, which can be attributed to the lack of novel antimicrobials entering the market [6]. As a result, various treatment approaches employing antivirulence substances have garnered considerable interest. The alternative therapeutic strategies, including antivirulence therapies, work by inhibiting bacterial virulence as opposed to antibacterials, which inhibit cell growth. Importantly, virulence factors are pathogenic in bacterial invasion and colonization but are not necessary for survival [7].

Antivirulence compounds have great potential [8] as they target nonessential genes for the survival of the bacteria and, as a result, (i) impose mild evolutionary pressure for the development of resistance, thus addressing the problem of the rapid rise in antimicrobial resistance; (ii) expand the range of pharmacological targets; and (iii) produce medicines with novel modes of action. Traditional medicine provides an unlimited opportunity to identify lead bioactive molecules from natural products for the development of novel antivirulence agents that can be paired with existing antibiotics to restore their efficacy against MRSA. Flavonoids, a class of phenolic compounds, present a promising option for developing antivirulence therapy in light of their role in regulating bacterial virulence [9]. Kuraridin—(E)-1-[2,4-dihydroxy-6-methoxy-3-(5-methyl-2-prop-1-en-2-ylhex-4-enyl) phenyl]-3-(2,4-dihydroxyphenyl)prop-2-en-1-one)—(Figure S1) is a flavonoid commonly found in the *Sophora flavescens* plant, which has been historically used in traditional medicine. This prenylated flavonoid has been reported to have potent beneficial pharmacological properties, including antiviral [10] and antitumor activity [11], tyrosinase inhibitory activity [12], glycosidase inhibitory activity [13], PLC_γ_1 inhibitory activity [14], Na^+^-glucose cotransporter SGLT inhibitory activity [15], and antibacterial actions [16]. Kuraridin has shown promise in various therapeutic applications, making it a subject of significant interest in contemporary studies. Kuraridin was recently identified as a potential natural anti-melanogenic agent that targets tyrosinase, a key enzyme in melanin production, to mitigate hyperpigmentation disorders caused by UV exposure [17]. It exhibits anti-inflammatory activity mediated via the inhibition of the AA-metabolizing enzymes COX/LOX [18]. This flavonoid demonstrates in vitro anti-reovirus activity via inhibiting viral replication and hemagglutination. Our previous study revealed for the first time that combining epicatechin gallate (ECG) with Kuraridin can enhance the in vitro antibacterial effects of gentamicin, fusidic acid, and vancomycin against MRSA. In a mouse infection model of pneumonia, this combined treatment resulted in a slight decrease in bacterial counts among MRSA-infected mice. Following this success in treating MRSA, combining Kuraridin and ECG may also be beneficial for tackling other drug-resistant bacterial infections [19]. In this study, we examined the effect of Kuraridin on three strains of MRSA regarding adherence to fibrinogen, adhesion, and internalization in human keratinocytes. We also investigated the potential of this flavonoid to protect the host during infection through the in vivo *Caenorhabditis elegans* infection model and to identify its target in MRSA. 

The USA300 pandemic clone of community-associated methicillin-resistant *S. aureus* (CA-MRSA) has recently become a leading cause of hospital-associated bloodstream infections (BSIs). USA300 is a predominant lineage in the USA and is a distant relation to hospital-associated methicillin-resistant *S. aureus* (HA-MRSA) that was not associated with healthcare settings [20,21]. However, recently, USA300 has been detected in hospital settings and is emerging as a prominent cause of nosocomial BSIs [21,22,23,24,25]. ST239 is acknowledged as a predominant HA-MRSA clonal type, which has exhibited persistent prevalence worldwide, demonstrating its adaptability [26,27,28,29,30]. In Iran, the overall prevalence of ST239 among patients was reported at 39.3%, with certain regions showing rates as high as 77.5% [31]. ST239 MRSA is common in numerous countries, especially in Asia. In Southeast Asia, MRSA ST239 was notably the most successful lineage in Brunei Darussalam, Cambodia, Indonesia, Lao People’s Democratic Republic (PDR), Malaysia, Myanmar, Thailand, Timor-Leste, Philippines, Singapore, and Vietnam. However, the prominent presence of ST239-III and ST241-III in this region during earlier observations has been replaced by the more antibiotic-susceptible MRSA strains such as ST22-IV and PVL-positive ST30-IV in recent years [32]. ST30 is one of the most commonly isolated lineages among CA-MRSA strains and is recognized as a pandemic clone due to its repeated isolation from every continent. Clonal complex 30 represents a successful *S. aureus* lineage, regardless of its methicillin resistance status [33]. In addition to skin and soft tissue infections (SSTIs), ST30 has been linked to an increased risk of infective endocarditis and bacteremia caused by *S. aureus* [34]. Therefore, the MRSA strain USA300 and two MRSA isolates representative of the clonal types ST30 and ST239 that are prevalent in Hong Kong were included in our study [35,36]. The sequence types and genotypes associated with distinct virulence factors of MRSA were described in our previous study [37].

## 2. Materials and Methods 

### 2.1. Bacterial Culture and Kuraridin 

The MRSA strains were grown at 37 °C with shaking at 200 rpm in BHI (Oxoid, Hampshire, UK) and Tryptic Soy broth (TSB) (Oxoid, Hampshire, UK) or on TS or blood agar. The MRSA strains were cultured in Mueller–Hinton broth to determine the minimum inhibitory concentration (MIC). *Escherichia coli* strain OP50 was grown in Luria–Bertani (LB) medium (Oxoid, Hampshire, UK). The HaCaT cell line was obtained from the Department of Microbiology, The Chinese University of Hong Kong. Kuraridin was purchased from SR Pharmasolutions (Hong Kong, China), and the structure of the compound was confirmed by mass spectrometry; the purity of Kuraridin was 96.19%, confirmed by HPLC. The MIC of Kuraridin was similar to our previously published results on *Sophora flavescens*-isolated Kuraridin [16]. All other chemicals were purchased from Sigma Chemical Company (St Louis, MO, USA). 

### 2.2. Evaluation of Antibiotic Susceptibilities of the MRSA Strains

The MICs of Kuraridin and Vancomycin against the MRSA strains were determined using the micro broth dilution method, according to the Clinical Laboratory Standards Institute (CLSI, 2019). 

### 2.3. Fibrinogen-Binding Assay

The effect of subinhibitory concentrations of Kuraridin on the adhesion of the MRSA strains to fibrinogen was evaluated via a fibrinogen-binding assay, as previously described [37]. Briefly, Polystyrene Costar 96-well (Corning, New York, NY, USA) plates were coated with 100 μL of BHI medium containing 10 μg/mL fibrinogen (Fg) (Sigma, St. Louis, MO, USA) at 4 °C overnight for 16 h. The plates were washed and blocked for 2 h at 37 °C with 2 mg/mL Bovine Serum Albumin (BSA). On the same day the 96-well plates were coated, a single colony was used to culture the bacteria overnight for 16 h in the BHI medium. The overnight culture was then diluted into 10 mL of fresh BHI and grown to the exponential phase in a shaking incubator; Kuraridin was added in measured amounts to each tube. The bacterial cells were harvested via centrifugation at 3000× *g* for 10 mins, washed twice with Phosphate-Buffered Saline (PBS), and resuspended in PBS to an OD_595 nm_ of 1.0. Then, 100 μL of bacterial cell suspension was added to each well after washing with PBS and incubated for 2 h at 37 °C. The cell suspension was aspirated, and the adherent bacterial cells were fixed with 25% (*v*/*v*) formaldehyde for 30 mins. After washing with PBS, 100 μL of crystal violet dye (12.5 mg/mL) was added and incubated for 10 mins. The plates were rewashed and air-dried. The absorbance of the plates was subsequently read at 595 nm by the DTX 880 Multimode Detector (Beckman Coulter, San Jose, CA, USA). The results are reported as the percentage of the adherence rate to solid-phase fibrinogen compared to the untreated bacteria [38].

### 2.4. Adhesion and Internalization Assays 

The adhesion and internalization assays were performed as previously described [37]. The human keratinocytes, HaCaT cells, were seeded at a density of 5 × 10^5^ cells per well and cultured in 24-well plates using Dulbecco’s modified Eagle’s medium (DMEM) (Gibco, Baltimore, MD, USA), supplemented with 10% FBS (Gibco, Baltimore, MD, USA) at 37 °C in a 5% CO_2_ atmosphere until the confluence was attained. The HaCaT cells were cultured for another 24 h to obtain matured keratinocytes. The keratinocytes were maintained in DMEM supplemented with 1% FBS for 1 h to avoid interference from serum components before bacterial infection. Overnight bacterial culture in BHI medium was diluted into 10 mL of fresh BHI and grown to log phase (OD_595_ = 0.4), along with the addition of Kuraridin in measured amounts to each tube. Bacterial cells were harvested via centrifugation at 3000× *g* for 10 mins and washed twice with PBS. The bacterial cells were resuspended in DMEM medium with 1% FBS and then added to keratinocytes at a multiplicity of infection (MOI) of 100 (5 × 10^7^) and co-cultivated with HaCaT cells at 37 °C in a 5% CO_2_ atmosphere for 90 mins. After that, the HaCaT cells were washed three times with PBS to remove any unbound bacteria and detached using 0.5% Trypsin (Gibco, Maryland, USA). The detached cells were lysed by incubation with 500 µL of 0.5% Triton X-100 per well for 15 mins. Colony Forming Units (CFUs) were enumerated via serial dilution, followed by plating the lysates onto horse blood agar plates and incubation overnight at 37 °C.

(1) $$Adherence\;of\;S.\;aureus\;to\;HaCaT\;cells\;in\;\%=\frac{Number\;of\;CFU\;recovered\;per\;well}{Number\;of\;CFU\;inoculated\;per\;well}\times 100$$

The bacterial infection of keratinocytes was conducted for 90 mins for the internalization assay. The medium was aspirated, and the HaCaT cells were washed three times with PBS to remove nonadherent bacteria. The HaCaT cells were then incubated with DMEM media supplemented with 100 µg/mL gentamycin (1000× in autoclaved distilled water), 10 µg/mL lysostaphin (1000× in autoclaved distilled water), and 1% FBS for 1 h at 37 °C to kill extracellular bacteria. The cells were then washed three times with PBS to remove the antimicrobials, detached, and lysed, as described for the adhesive assay.

(2) $$Internalization\;of\;S.\;aureus\;to\;HaCaT\;cells\;in\;\%=\frac{Number\;of\;CFU\;recovered\;per\;well}{Number\;of\;CFU\;recovered\;per\;well\;in\;control}\times 100$$

### 2.5. Determination of Biofilm Biomass by Crystal Violet Staining 

The biofilm biomass was determined following the previously reported method [39] with slight modifications. A single colony of each of the overnight bacterial cultures was inoculated in 5 mL of BHI, incubated overnight for 16 h, and then diluted to 1:100 in BHI supplemented with glucose (1% *w*/*v*). An aliquot of 200 µL of each diluted culture was added to each well of a flat-bottom 96-well microtiter plate (Costar, Boston, MA, USA) and incubated statically at 37 °C for 24 h in a humidified chamber. The culture medium was removed, and the plates were washed thrice with PBS to remove the floating cells. The biofilms were stained with 200 µL of 0.5% crystal violet (prepared in 10% ethanol) for 15 min at room temperature. After staining, the plates were gently washed with PBS three times and dried at room temperature. Then, 100 µL of 95% ethanol was added to each well and incubated for 15 min to dissolve the biofilms. The absorbance was measured at 492 nm using a Ledetect 96 plate reader (Labexim Products, Lengau, Austria).

### 2.6. Confocal Laser Scanning Microscopy (CLSM) Analysis of Biofilms

The bacterial cultures were prepared as described above. The bacterial cultures were then diluted to 1:100 in BHI supplemented with glucose (1% *w*/*v*), and 500 µL was added to each well of the Nunc LabTek II (Sigma, St. Louis, MO, USA) 8-well chamber and incubated statically at 37 °C for 24 h in a humidified chamber. The chambers were washed three times with PBS, and the biofilms were fixed with 4% formalin for 15 mins. After formalin fixation, biofilms were washed with PBS and stained with the LIVE/DEAD BacLight Bacterial Viability Kit (Invitrogen, Waltham, MA, USA), following the manufacturer’s instructions. Slides were then mounted using ProLong Diamond Antifade Mountant (Invitrogen, MA, USA). Confocal laser scanning microscopy was performed using the CarlZeiss LSM880 Laser Confocal Microscope (CarlZeiss, Oberkochen, Germany), sequentially scanning with a 488 nm argon laser for excitation. The emitted fluorescence of Syto9 was recorded within the range of 505–530 nm. The Z-stacks from the bottom of the biofilm were captured every 10 μm section at different areas in the well for the analysis of the thickness of the biofilms. The Carl Zeiss Zen3.2 (blue edition) was used to analyze the images [40].

### 2.7. C. Elegans Husbandry

The *C*. *elegans* Bristol N2 wild-type strain was used throughout this study. The nematodes were maintained at 25 °C on nematode growth medium (NGM) and fed *E. coli* OP50. The gravid nematodes were age-synchronized by bleaching with alkaline hypochlorite to isolate embryos. 

### 2.8. Evaluation of Kuraridin Furnished Protection of Infection Caused by the MRSA Strains in the C. Elegans Model 

The effect of Kuraridin on the *C. elegans* infection model was evaluated in a liquid medium in 96-well plates, according to Kong et al. [41]. The liquid screening medium consisted of 80% M9 buffer, 20% *S. aureus* 16 h overnight culture grown in BHI, and 10 μg/mL cholesterol. Kuraridin was added to the medium at 200 µg/mL. The assay components were mixed evenly, and 120 μL of the medium was added into each well in a 96-well plate. Twenty age-synchronized L4 stage nematodes were transferred into each well. To eliminate interference in scoring surviving worms due to reproduction, 30 μL of 0.6 mM Fluorodeoxyuridine (FUDR) to a final concentration of 0.12 mM was added [42]. Wells containing *C. elegans*, M9 buffer, and *E. coli* OP50 served as the negative control. The positive control wells included 1 µg/mL of vancomycin. The test was conducted in triplicate wells. Worm survival was scored manually using a Nikon SMZ1000 (Nikon, Melville, NY, USA) microscope every 24 h for 120 h (5 days). 

### 2.9. Microscopy of the C. Elegans for Evaluation of Intestinal Lumen 

After 24 h of infection in the liquid kill assay, the nematodes were anesthetized via treatment with 10 uL of 10 mM NaN_3_ and placed on an agar pad for microscopic observation at 100× magnification.

### 2.10. Molecular Docking Using Swiss Dock

In connection with identifying the protein target, the optimal ligand/protein configuration was determined, and the free energy of binding of Kuraridin was predicted via a molecular docking study. Molecular docking was performed using SwissDock in the CHARMM force field with EADockDSS24 [43]. The three-dimensional crystal structure 1T2W, which represents the SrtA enzyme of SA and has been used in previous in silico screenings of sortase inhibitors [44,45], was selected for this study and retrieved from the Protein Data Bank (http://www.rcsb.org/) (accessed on 14 November 2023). 1T2W has a C184A mutation in the active site; therefore, the residue Ala-184 was mutated to wild-type Cys and the neighboring residues were optimized using Chimera (Cys-184_1T2W). To improve the accuracy of the docking calculations for each MRSA isolate used in the study and to address strain-specific variations in SrtA, which may occur in different strains, the three-dimensional structure models representative of SrtA in USA300, ST30, and ST239 were prepared with the SWISS-MODEL program using PDB: 1T2W as a template. The protein molecule was loaded in the Chimera 1.14 for molecular docking purposes, and a single polypeptide chain A was selected for the docking study to reduce the CPU time required. The receptor polypeptide chain was prepared for docking using the Dock Prep module. The ligand and solvent molecules were deleted, and the incomplete side chains were added using the Dunbrackrotamer library. Hydrogens were added considering the H bonds (slow), and protonation states for histidine were added. The charges were assigned to the standard residues using AMBERff14SB, and Gasteiger charges were added to other residues. The docking study was performed in the Swiss Dock web-based server. The docking was performed using the default parameters, and a blind docking approach was used. The results were visualized in the View Dock tool [46] of Chimera. The docking of the peptide LPETG, which is the natural substrate of SrtA, was performed against each respective protein structure and served as the control. 

### 2.11. SrtA Activity Inhibition Assay

The inhibitory activity of Kuraridin on the SrtA enzyme was determined by quantifying the increase in fluorescence intensity upon cleavage of a synthetic peptide substrate, according to a previously documented procedure [47], with slight modifications. Briefly, the reactions were performed in a volume of 300 µL containing Tris-HCl 50 mM, CaCl_2_ 5 mM, NaCl 150 mM, pH 7.5, recombinant SrtA enzyme (BPS Bioscience, San Diego, CA, USA) 5 µM, fluorescent peptide substrate (Bacterial Sortase Substrate III, Abz/DNP Abz–LPETG–K(Dnp)–NH2, ANASPEC) 10 µM, and Kuraridin at the concentrations 25 mM, 50 mM, 75 mM, 100 mM, and 125 mM. The stock solution of each test compound was diluted with sterile distilled water before use. Blanks contained all of the above-mentioned substances except for the Kuraridin. Reactions were carried out for 1 h at 30 °C and analyzed fluorometrically using EnSpire Multimode Plate Reader (Perkin Elmer, Hopkinton, MA, USA) at 320 nm for excitation and 420 nm for recordings.

### 2.12. Statistical Analysis

Statistical analyses and significance, measured using one-way ANOVA followed by Tukey’s post hoc analysis, were performed using GraphPad PRISM software version 9.0 (GraphPad Software, San Diego, CA, USA). In all comparisons, *p* < 0.05 was considered statistically significant.

## 3. Results

### 3.1. Minimum Inhibitory Concentrations (MICs) 

The MICs of Kuraridin and vancomycin against the strains are given in Table 1. 

After establishing the MIC values for Kuraridin against the representative MRSA strains, growth curves were performed to reveal the impact of subinhibitory concentrations of Kuraridin on bacterial growth. The growth of the MRSA strain USA300 was measured in the presence of Kuraridin at subinhibitory concentrations that were dilutions of the MIC: 2 µg/mL (1/8 MIC), 1 µg/mL (1/16 MIC), and 0.5 µg/mL (1/32 MIC). The growth of the MRSA strains ST30 and ST239 was also measured in the presence of Kuraridin at subinhibitory concentrations that were dilutions of the MIC: 1 µg/mL (1/8 MIC), 0.5 µg/mL (1/16 MIC), and 0.25 µg/mL (1/32 MIC). These concentrations did not impair the growth of the respective strains (Figure S2).

### 3.2. Adherence to Fibrinogen in the Presence of Kuraridin 

**Figure 1:**
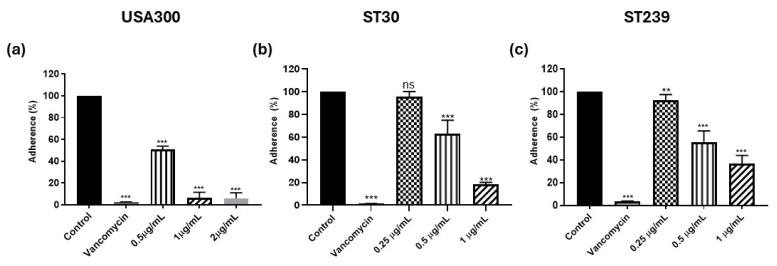
The adherence of MRSA USA300 (**a**), ST30 (**b**), and ST239 (**c**) to solid-phase fibrinogen in the presence of Kuraridin at subinhibitory concentrations of 1/8, 1/16^,^ and 1/32. The error bars represent the standard deviation of the mean values (*n* = 9). Significance was obtained by one-way ANOVA followed by the Tukey post hoc test. The *p*-values were obtained by comparison between the mean of the treatments relative to the control and are coded as ** *p* < 0.01, and *** *p* < 0.001. ns signifies no significant difference relative to the control.

Even though fibrinogen may dramatically reduce bacterial infections by acting as an antibacterial host defense component, SA has successfully developed strategies to interact with the host fibrinogen to shift the scales toward infection pathogenesis. These virulence factors have allowed MRSA to both avoid the antibacterial effects brought on by fibrin(ogen) and “hijack” the coagulation system of the host to elevate the pathogenicity of the pathogen. To investigate whether Kuraridin hinders the binding of the MRSA strains to fibrinogen, we performed the fibrinogen-binding assay in which the adhesion of bacteria to solid-phase fibrinogen coated on polystyrene plates was quantified by the absorbance following staining of the bacteria with crystal violet. The adherence of the MRSA strains was measured in the presence of Kuraridin at subinhibitory concentrations of the MICs. Vancomycin (1 µg/mL) was taken as the bactericidal control. We observed a significant reduction in the adherence of the MRSA strains when treated with Kuraridin. The fold change in the adherence of the USA300 was 93.84, 51.37, and 13.08 upon treatment with Kuraridin at 2 µg/mL, 1 µg/mL, and 0.5 µg/mL, respectively (Figure 1a). The fold change in adherence of strain ST30 was 81.58 and 36.75 upon Kuraridin treatment at 1 µg/mL and 0.5 µg/mL. No significant change in adherence was observed when strain ST30 was treated with 0.25 µg/mL of Kuraridin (Figure 1b). In the case of strain ST239, the fold changes in adherence were observed to be 62.90, 44.18, and 7.654 upon treatment with Kuraridin at 1 µg/mL, 0.5 µg/mL, and 0.25 µg/mL, respectively (Figure 1c).

### 3.3. Adhesion and Internalization in HaCaT Cells in the Presence of Kuraridin

**Figure 2:**
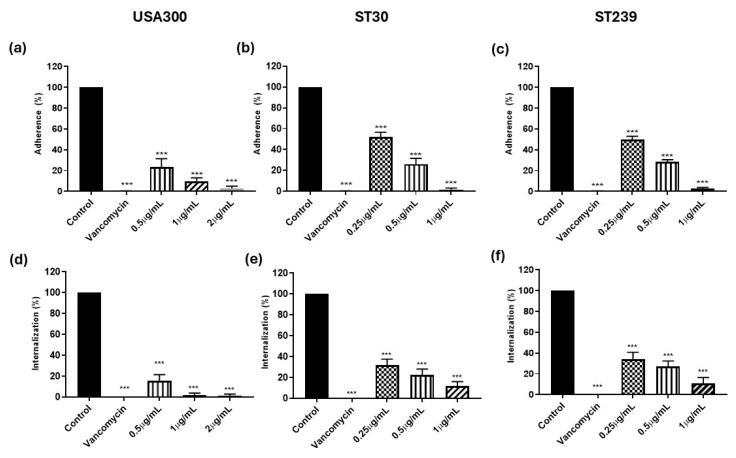
The adherence of the strains USA300 (**a**), ST30 (**b**), and ST239 (**c**) to the HaCaT keratinocytes in the presence of the Kuraridin at subinhibitory concentrations of 1/8, 1/16^,^ and 1/32. The internalization of the strains USA300 (**d**), ST30 (**e**), and ST239 (**f**) to the HaCaT keratinocytes in the presence of Kuraridin at subinhibitory concentrations of 1/8, 1/16^,^ and 1/32. The error bars represent the standard deviation of the mean values (*n* = 3). Significance was obtained by one-way ANOVA followed by the Tukey post hoc test. The *p*-values were obtained by comparing the mean of the treatments relative to the control. *** signifies *p* < 0.001.

The microbial surface components recognizing adhesive matrix molecules (MSCRAMMs) of SA facilitate their attachment to host ECM substances, and this binding ability is directly linked to the pathogenicity of staphylococci since the invasion of host cells depends on the adhesion of SA to ECM or plasma proteins. To determine the impact of Kuraridin on the adherence and internalization of MRSA strains by keratinocytes, the interaction of the MRSA strain with HaCaT keratinocytes in the presence of 2, 1, or 0.5 μg/mL in the case of USA300 and 1, 0.5, and 0.25 μg/mL in case of ST30 and ST239 was studied. The HaCaT keratinocytes were seeded at a concentration of 5 × 10^5^ cells per well and cultured at 37 °C in a 5% CO_2_ atmosphere for 24 h in Dulbecco’s modified Eagle’s medium (DMEM, Gibco, Grand Island, NY, USA) supplemented with 10% FBS (Gibco, Grand Island, NY, USA) until confluent and allowed to grow for another 24 h before bacterial inoculation to attain maturation. The experiments were conducted with an MOI of 100 and a co-cultivation time of 90 mins. Adherence of the respective MRSA strains without adding the Kuraridin was taken as a control. Significant (*p* < 0.001) fold reductions of 90.14, 76.58, and 62.57 when treated with 2, 1, or 0.5 μg/mL, respectively, in the adherence of the strain USA300 to HaCaT cells were observed (Figure 2a). The fold reduction in the adherence of strain ST30 to HaCaT cells was significant (*p* < 0.001), measuring 98.24, 73.85, and 47.80 when treated with 1, 0.5, or 0.25 μg/mL, respectively (Figure 2b). The fold reduction in adherence of strain ST239 to HaCaT cells was 97.17, 71.32, and 50.28 when treated with 1, 0.5, or 0.25 μg/mL, respectively (Figure 2c). The fold reduction in the internalization of the strain USA300 to HaCaT cells was 98.02, 84.13, and 75.79 when treated with 2, 1, or 0.5 μg/mL, respectively (Figure 2d). The fold reduction in the internalization of strain ST30 to HaCaT cells was 88.20, 77.53, and 67.98 when treated with 1, 0.5, or 0.25 μg/mL, respectively (Figure 2e). The fold reduction in the internalization of strain ST239 to HaCaT cells was 88.95, 72.67, and 65.7 when treated with 1, 0.5, or 0.25 μg/mL, respectively (Figure 2f). A dose-dependent and significant (*p* < 0.001) reduction in adhesion and internalization in HaCaT cells throughout all three strains was observed.

### 3.4. Biofilm Formation in the Presence of Kuraridin

**Figure 3:**
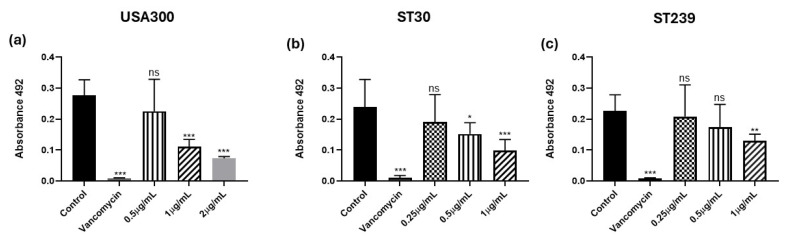
The dose–response plots of Kuraridin on biofilm formation tested against MRSA strains USA300 (**a**), ST30 (**b**), and ST239 (**c**). The error bars represent the standard deviation of the mean values (*n* = 3). Significance was obtained by one-way ANOVA followed by the Tukey post hoc test. The *p*-values were obtained by comparing the mean of the treatments concerning the control and are coded as * *p* < 0.05, ** *p* < 0.01, and *** *p* < 0.001. ns depicts no significant difference relative to the control.

To investigate whether Kuraridin impairs MRSA biofilm formation, we evaluated Kuraridin at 1/8, 1/16, and 1/32 MIC. The formation of biofilm upon treatment with Kuraridin was assessed by crystal violet staining (Figure 3). Biofilm formation was significantly inhibited by a 1/8 MIC of Kuraridin concentration in USA300 (*p* < 0.001), ST30 (*p* < 0.001), and ST239 (*p* < 0.01). The inhibition of biofilm formation occurred in a dose-dependent manner. However, the decrease in biofilm formation was insignificant in the USA300 and ST30 strains when treated with a 1/32 MIC and in the ST239 strain when treated with 1/16 and 1/32 MICs. 

**Figure 4:**
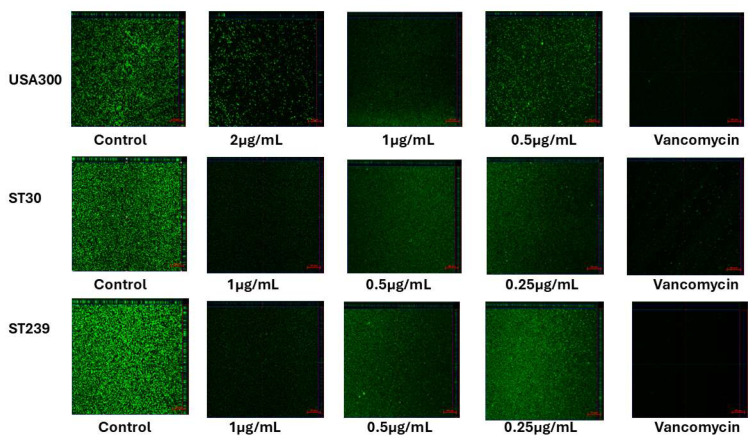
Confocal images of the MRSA strains USA300, ST30, and ST239 biofilm formation in the presence of Kuraridin at 1/8, 1/16, and 1/32 MICs, respectively. The central panels represent the x–y plane, and the top and right-side panels represent the x–z and y–z planes, respectively. The scale bar represents 50 μm.

The biofilm formation of the MRSA strains was evaluated using CSLM. The biofilms were stained with Syto9 at 24 h before CSLM analysis. The USA300, ST30, and ST239 confocal images are shown in Figure 4. Compared to the samples treated with Kuraridin or vancomycin, the USA300, ST30, and ST239 untreated biofilms showed confluent growth and increased biovolume (xy plane). Following treatment with MICs of 1/8, 1/16, and 1/32, the biofilm development was gradually reduced in the MRSA strains. The biofilm of the untreated samples exhibited multidirectional expansion throughout 24 h, both in the xy (increase in surface coverage) and z planes (increase in thickness). After treatment with Kuraridin at a 1/8 MIC, the z-projection of the xy-stacks demonstrated differences in the biofilm thickness between USA300, ST30, and ST239. The untreated samples form thicker, more compact biofilms than their Kuraridin- or vancomycin-treated counterparts.

### 3.5. Molecular Docking Studies of Kuraridin with SrtA Protein 

**Figure 5:**
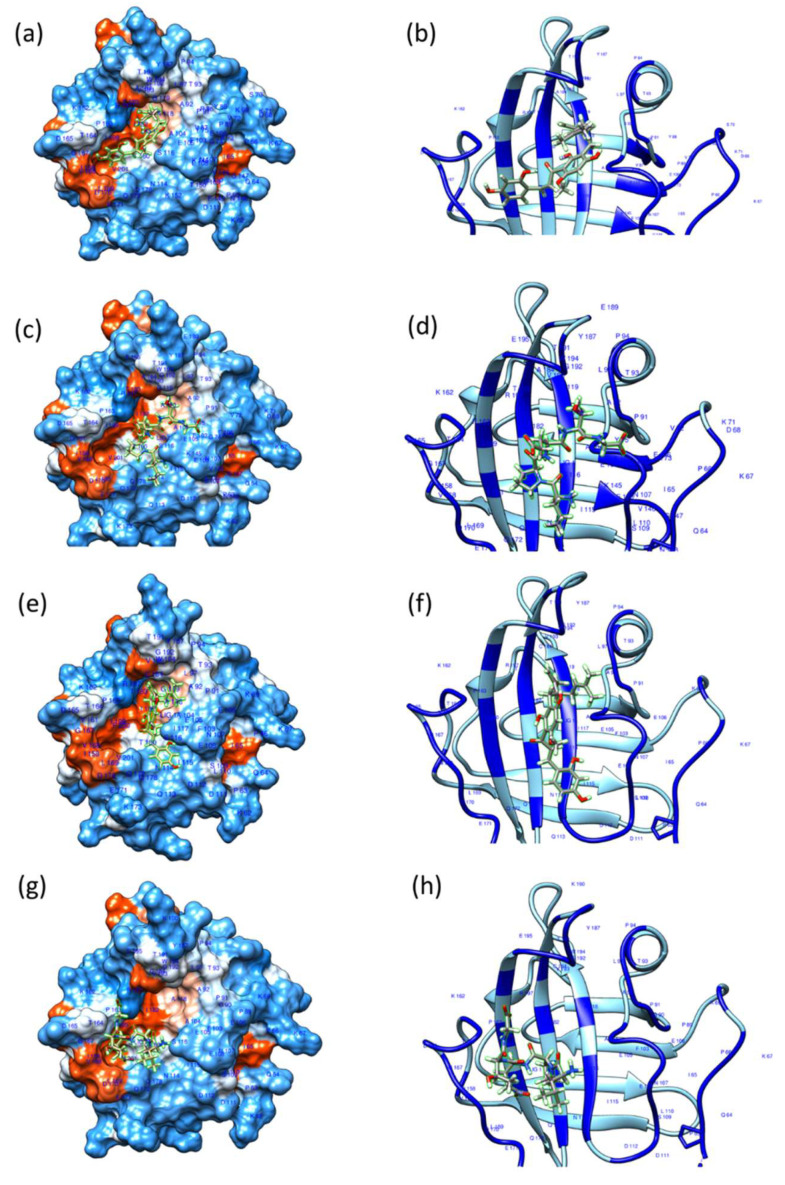
Docking configurations illustrate the most energetically favorable binding mode of the ligands Kuraridin (**a**,**b**,**e**,**f**) and the peptide LPETG (**c**,**d**,**g**,**h**) to Sortase A, as demonstrated by Chimera (1.14). The ligands are represented as stick models. The ligand binding site in the SrtA apo structure (PDB ID: 1T2W) (**a**,**c**) and in the SrtA apo structure (Cys-184_1T2W) (**e**,**g**) are shown as a surface in the left panels and as a ribbon (**b**,**d**,**f**,**h**) in the right panels, with residues within 5 Å of the bound ligand highlighted in deep blue.

**Figure 6:**
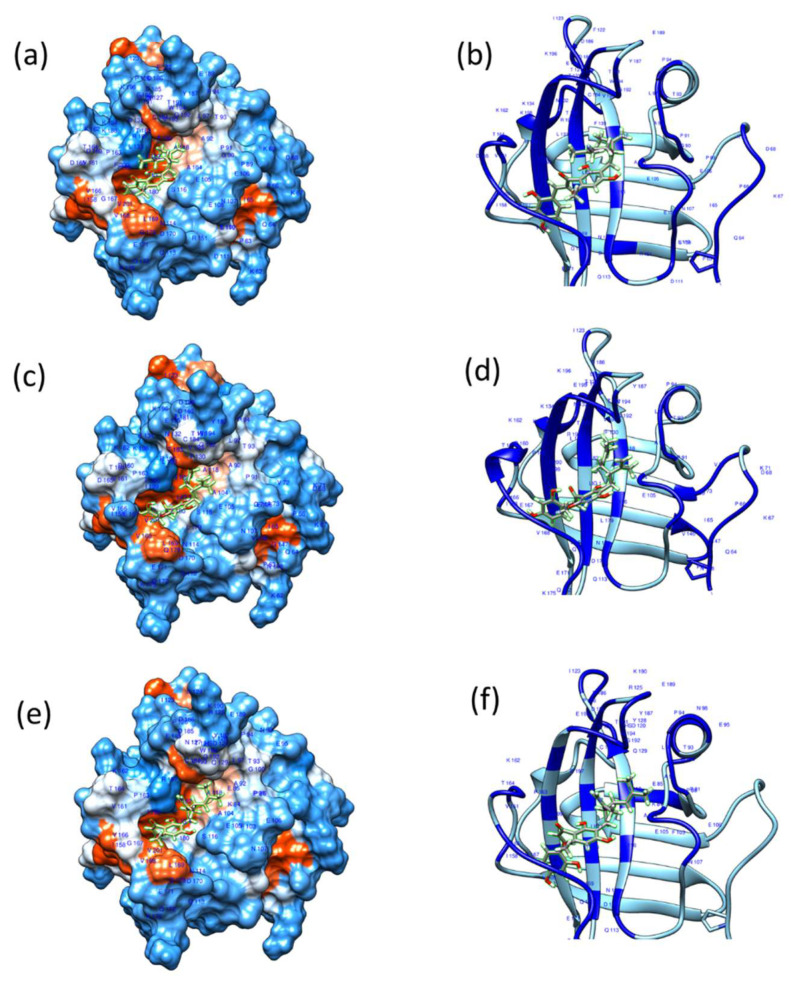
Chimera illustrating the docking configurations of the most energetically favorable binding mode for the ligand Kuraridin into Sortase A (1.14). Three-dimensional structural models of USA300 (**a**,**b**), ST30 (**c**,**d**), and ST239 (**e**,**f**) of Sortase A were created using the SWISS-MODEL program, with PDB: 1T2W serving as a template. The ligand is depicted as a stick model. In the SrtA apo structures (**a**,**c**,**e**), the ligand binding site is shown as a surface in the left panels and as a ribbon in the right panels (**b**,**d**,**f**), with residues within 5Å of the bound ligand highlighted in deep blue.

Based on the findings that Kuraridin suppresses the binding ability of the three MRSA strains to fibrinogen and the HaCaT cells and the biofilm formation, we investigated if Kuraridin may bind to the SrtA enzyme and interfere with its enzymatic activity. We performed docking experiments using SwissDock (http://www.swissdock.ch/) (accessed on 14 November 2023). Polypeptide chain A of the crystal structure of SrtA from *SA*, PDB Id 1T2W, with a resolution of 1.8 Å, was selected for docking calculation. The crystal structure of SrtA (1T2W) is complexed with its natural substrate, the LPETG sequence. The peptide LPXTG binds to SrtA through a large groove leading into the active site. The residues in strands 4 and 7 from the groove floor and the surface loops that connect strand 6 to strand 7 (β6/β7 loop), strand 7 to strand 8 (β7/β8 loop), strand 3 to strand 4 (β3/β4 loop), and strand 2 to helix H1 (β2//H1 loop) form the groove walls (Figure 5). The loop β6/β7 is reported to be essential for enzyme catalysis function, as a mutation in the loop β6/β7 region impairs enzyme activity and alters substrate recognition specificity [48,49,50]. Molecular docking calculations indicated that Kuraridin binds to the active site groove of SrtA proteins 1T2W and Cys-184_1T2W in a similar way to the natural substrate LPETG. Figure 5 shows the most energetically favorable binding mode for Kuraridin and LPETG to SrtA. Similarly, docking calculations showed that Kuraridin bound to the active site groove of the homology-modeling-derived SrtA proteins of the MRSA strains USA300, ST30, and ST239 (Figure 6). Figure S3 shows the docking of the natural substrate LPETG peptide to the active site groove of the homology-modeling-derived SrtA proteins of the MRSA strains USA300, ST30, and ST239. Table 2 shows the ΔG (kcal/mol) and the full fitness score of the docking experiments. The LogIC50 of Kuraridin against SrtA was 1.699 ± 0.055 µM. The statistical analysis was conducted utilizing GraphPad Prism 9. LogIC50 was established by translating the X value and employing a nonlinear fit of log (inhibitor) against the response-variable slope (four parameters).

### 3.6. Evaluation of Kuraridin Toxicity and Protection Against Infection Caused by MRSA in C. Elegans Model

**Figure 7:**
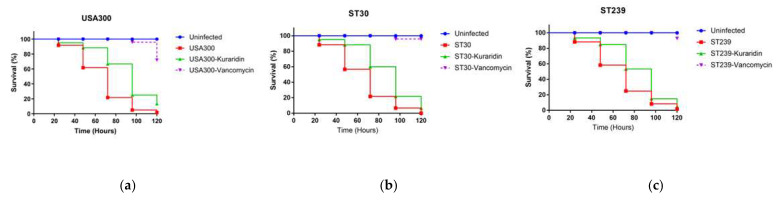
Survival curves depicting the effect of Kuraridin in *C. elegans* infected with USA300 (**a**), ST30 (**b**), and ST239 (**c**). Kaplan–Meier survival analysis was used to prepare survival curves, and the data were compared with untreated worms. A *p*-value < 0.05 was considered significant.

**Figure 8:**
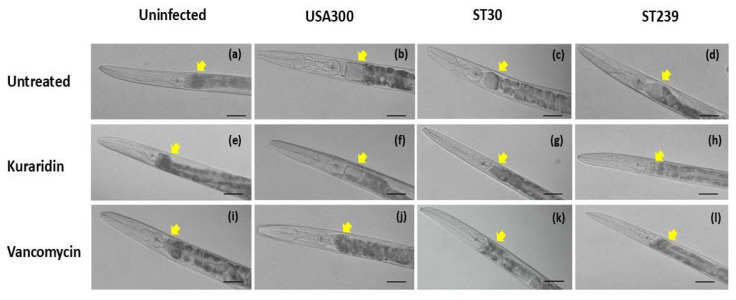
Micrographs showing the intestinal lumens of the *C. elegans*. The yellow arrows indicate the intestinal lumen. Distention of lumens was not observed in uninfected (**a**), Kuraridin-treated (**e**), and vancomycin-treated (**i**) *C. elegans*. The intestinal lumens of the nematodes infected with USA300 (**b**), ST30 (**c**), and ST239 (**d**) were distended, whereas the distention was observed to be reduced in the infected nematodes after being treated with Kuraridin, as shown in (**f**,**g**,**h**), respectively. The lumens of the nematodes treated with vancomycin (**j**,**k**,**l**) were comparable to the uninfected nematode. The scale bar represents 100 μm.

*C. elegans* is an alternative model for assessing novel antimicrobial drugs’ in vivo toxicity and effectiveness. This nematode is a rapid and affordable experimental host compared to more expensive mammalian models [51]. The efficacy of Kuraridin in protecting the nematodes against the MRSA strains was evaluated. Figure S4 shows the timeline of the infection. Compared to the negative control group, nematodes treated with Kuraridin or vancomycin at 1 μg/mL showed a significantly higher survival rate after MRSA infection (Figure 7). In parallel, the microscopy revealed that, on day 1 (Figure 8), the nematodes infected with USA300 (Figure 8b), ST30 (Figure 8c), and ST239 (Figure 8d) had distended intestinal lumens. The intestinal lumens of worms treated with Kuraridin showed a reduction in intestinal distention, and those treated with vancomycin were comparable to the uninfected nematodes, supporting our findings from the survivability experiments. Vancomycin, which functions as a bactericidal drug, protected *C. elegans* against infection to a more significant (*p* < 0.0001) extent than Kuraridin, which has antivirulence effects without affecting growth. The lack of mortality (Figure S5) and obvious lack of morphological damage (Figure 8e) following the administration of Kuraridin at the dose of 200 μg/mL shows that the Kuraridin is not toxic. This information supports our earlier in vitro findings that Kuraridin is a noncytotoxic anti-MRSA agent [16].

## 4. Discussion

The rise in multi-drug-resistant bacterial infections has raised alarm among health organizations worldwide and expedited the requirement for new potent treatments [52]. In this regard, antivirulence therapeutics present prospective cutting-edge treatment solutions that differ from conventionally prescribed antibiotics in their method of action. Here, we demonstrate that Kuraridin decreases MRSA virulence in an in vivo model of *C. elegans* without impeding bacterial growth, strongly suggesting that Kuraridin, at subinhibitory concentrations, has antivirulence properties and operates in a manner different from that of the antibiotic vancomycin. The antibacterial effects of Kuraridin are significant, particularly against MRSA. Weng et al., 2023 showed that Sophoraflavanone G and Kurarinone, two of the most abundant lavandulylated flavonoids in *S. flavescens*, exhibited antibacterial activity against *S. aureus. Sophoraflavanone* G exhibited an MIC90 of 3.9 µg/mL against MRSA USA300, MSSA ATCC 25923, and MSSA ATCC 29213. The MIC90 value of Kurarinone against MRSA USA300 and MSSA ATCC 29213 was 7.8 µg/mL, whereas this value was 3.9 µg/mL against MSSA ATCC 25923. Kurarinone’s anti-MRSA activity was comparable to Kuraridin’s. However, it must be noted that the USA300 strain used in our study may vary from that used by Weng et al., 2023. Naringenin, another flavonoid isolated from *S. flavescens*, did not exhibit significant antibacterial activity [53]. Notably, our previous study reported that Kuraridin showed no toxicity on human peripheral blood mononuclear cells (PBMCs) at concentrations up to 64 μg/ml, while Sophoraflavanone G inhibited over 50% of cellular activity at 4 μg/ml or higher concentrations. Although possessing a higher antibacterial activity, this toxicity of Sophoraflavanone G at therapeutic concentrations indicates that Kuraridin is potentially a safer alternative among *S. flavescens* flavonoids [16]. Sohn et al. evaluated the antimicrobial and cytotoxic activity of Kuraridin and three other flavonoids, namely, 5-methylsophoraflavanone B, Kurarinone, and Sophoraflavanone G isolated from *S. flavescens.* Only Kuraridin exhibited antifungal activity, with an MIC of 25µg/mL against *Candida albicans* ATCC 10231, which was used as a representative fungus of candidiasis. Strong antibacterial activity was exhibited by Kuraridin (MIC 20µg/mL) against the bacterial strains *E. coli* KCTC 1924, *Salmonella typhimurium* KCTC 1926, *Staphylococcus epidermis* ATCC 12228, and *S. aureus* KCTC 1621. Kurarinone also exhibited activity against Gram-positive bacteria, with an MIC of 20µg/mL against *E. coli, S. epidermis,* and *S. aureus,* and an *MIC of 50* µg/mL against *S. typhimurium*. No antimicrobial activity was observed for 5-methylsophoraflavanone B. Kuraridin exhibited low cytotoxicity, with an IC50 value of 37.8 µg/mL in the HepG2 cell line. Considering its low cytotoxicity and potent antimicrobial activity, Kuraridin could be used to treat microbial infections [54]. 

The capacity of MRSA to express a wide variety of virulence factors at various phases of host colonization and infection is closely connected to the organism’s pathogenicity. MRSA preferentially expresses surface proteins necessary for attachment to host-extracellular-matrix molecules in the early stages of infection. These microbial surface components recognize adhesive matrix molecules (MSCRAMMs), like fibrinogen-binding proteins and clumping factors, and are crucial mediators of early bacterial attachment [55]. 

Our findings indicate that Kuraridin hinders MRSA during the early adhesion phases without inhibiting growth. The transpeptidase activity of SrtA is responsible for covalently anchoring several virulence-associated surface proteins to bacterial cell wall peptidoglycan. By regulating the capacity of the bacterium to attach to host tissue and abiotic surfaces, the SrtA plays a crucial part in the development of infection [56]. Consequently, this enzyme is a prospective pharmaceutical target that has the potential to significantly lessen bacterial virulence [32]. Here, we demonstrate that Kuraridin treatment of the three MRSA strains results in a decreased capacity to bind fibrinogen and HaCaT cells, indicating that Kuraridin modulates surface proteins required in bacterial adhesion. The effect of the Kuraridin on the binding to the fibrinogen was dose-dependent and strain-dependent. A docking study predicted that Kuraridin binds to human fibrinogen, which may also play a role in reducing the adhesion of MRSA strains to solid-phase fibrinogen (Figure S6).

Docking studies predicted that the Kuraridin binds to the active site of the SrtA, inhibiting the activity of SrtA transpeptidase. SrtA catalyzes the covalent anchoring of surface proteins to the cell wall of SA [57,58]. The surface proteins include fibrinogen-binding proteins, clumping factors (ClfA and ClfB), and fibronectin-binding proteins A and B (FnbA and FnbB) and are involved in pathogenesis. Thus, mutants that lack a functional *srtA* gene or inhibit the SrtA enzyme will be defective in establishing infections as they cannot display surface proteins [56].

The function of Kuraridin is novel as it inhibits SrtA, which impacts the surface proteins and secreted proteins of SA. We hypothesize that a coating of Kuraridin on medical equipment will be able to effectively inhibit the growth of MRSA biofilms, a useful application in light of biofilm recalcitrance and the challenge of removing them from medical devices. Polyphenol-based coatings and other natural compounds with known therapeutic potential but minimal immunogenicity and toxicity constitute an environmentally friendly alternative to pharmaceuticals [59,60,61].

## 5. Conclusions

In conclusion, our study elucidates the significant potential of Kuraridin as an antivirulence agent against MRSA. The findings reveal several critical mechanisms by which Kuraridin operates. (i) Kuraridin exhibits remarkable antibiofilm properties that inhibit the formation of robust bacterial biofilms, which are crucial for MRSA’s virulence and ability to persist in hostile environments. (ii) Kuraridin has been observed to reduce binding to fibrinogen, a protein that facilitates the establishment of an infection within the host. This reduction is pivotal as it impedes the ability of MRSA to adhere to host tissues and initiate infection. (iii) Kuraridin decreases both adhesion and internalization, which are fundamental for the propagation of infection. In silico docking experiments and enzyme inhibition studies showed that this is likely due to its direct binding to the SrtA enzyme, which plays a vital role in bacterial adhesion. Kuraridin offers a sizable degree of protection against MRSA infections without being toxic to the nematode *C. elegans*. This supports our earlier in vitro results that Kuraridin is a noncytotoxic anti-MRSA agent. Our findings highlight Kuraridin as a promising candidate for combating MRSA. 

## Figures and Tables

**Table 1:** Minimum inhibitory concentrations (MICs) of Kuraridin and vancomycin against MRSA strains.

MRSA Strains	MIC Kuraridin (µg/mL)	MIC Vancomycin (µg/mL)
USA300	16	1
ST30	8	1
ST239	8	1

**Table 2:** The ΔG (kcal/mol) and full fitness scores of Kuraridin and LPETG peptide calculated with Swiss Dock.

Target and Ligand	ΔG (kcal/mol)	Full Fitness Score
1T2W-K	−8.1571	−1379.1876
1T2W-LPETG	−9.6109	−1358.3839
Cys-184_1T2W-K	−8.4931	−1386.3301
Cys-184_1T2W-LPETG	−9.5649	−1356.9520
* USA300- SRTA-K	−8.1275	−1276.1605
* USA300- SRTA-LPETG	−9.4921	−1252.7101
* ST30 SRTA-K	−8.6170	−1311.6985
* ST30 SRTA-LPETG	−10.0452	−1292.4149
* ST239 SRTA-K	−8.2518	−1276.3574
* ST239 SRTA-LPETG	−9.5763	−1229.8800

Note: * depicts the protein prepared by homology modeling using SWISS-MODEL.

## Data Availability

The original contributions presented in this study are included in the article/Supplementary Materials. Further inquiries can be directed to the corresponding author.

## Supplementary Materials

The following supporting information can be downloaded at: https://www.mdpi.com/article/10.3390/biomedicines13030564/s1, Figure S1: Kuraridin 2D structure; Figure S2: Growth kinetics at different concentrations of Kuraridin at 1/8th, 1/16th, and 1/32nd MIC during 24 h against MRSA USA300 (a), ST30 (b), and ST239 (c).; Figure S3: Docking poses of the most energetically favorable binding mode of the natural substrate LPETG into the Sortase A as visualized by Chimera (1.14). Three-dimensional structure models of USA300 (a, b), ST30 (c, d), and ST239 (e, f) of SortaseA were prepared with the SWISS-MODEL program, using the PDB ID: 1T2W2 as a template. The ligand is shown as a stick model. The ligand binding site in the SrtA apo structures(a,c,e) are shown as surface in the panels on the left side and as ribbon (b, d, f) in the panels on the right side with the residues with 5Å of the bound ligand are highlighted in deep blue; Figure S4: Timeline of in vivo study to determine the effect of Kuraridin on the survivability of *C. elegans* infected with the MRSA strains. The figure has been produced using Servier Medical Art (http://smart.servier.com/ accessed on 31 May 2022); Figure S5: Survival of *C. elegans* at the concentration of 200 µg/mL of Kuraridin. Kaplan–Meier survival analysis was used to prepare survival curves, and the data were compared with untreated worms. No significant difference (*p* > 0.9999) in mortality was observed between untreated and Kuraridin-treated nematodes; Figure S6: The visualization of the most energetically favorable binding mode of the ligand Kuraridin into the human Fibrinogen (PDB ID: 1RF1). The maximum affinity with G of −8.5322.kcal/mol and d Fullfitness score of −3275.3123was calculated using SwissDock. The ligand is shown as a stick model. For clarity, only interacting important residues are displayed. The residues within 5Å of the bound ligand are highlighted in deep blue.
