# Supplementary material for: Antivirulence Properties of Kuraridin Against Methicillin-Resistant Staphylococcus aureus (MRSA)
Source: Biomedicines. 2025 Feb 24;13(3):564. doi: 10.3390/biomedicines13030564 (PMC11940505; doi:10.3390/biomedicines13030564)
Supplement: Supplementary file 1 [file biomedicines-13-00564-s001.zip › biomedicines-3465937-supplementary.pptx]

## Slide 1
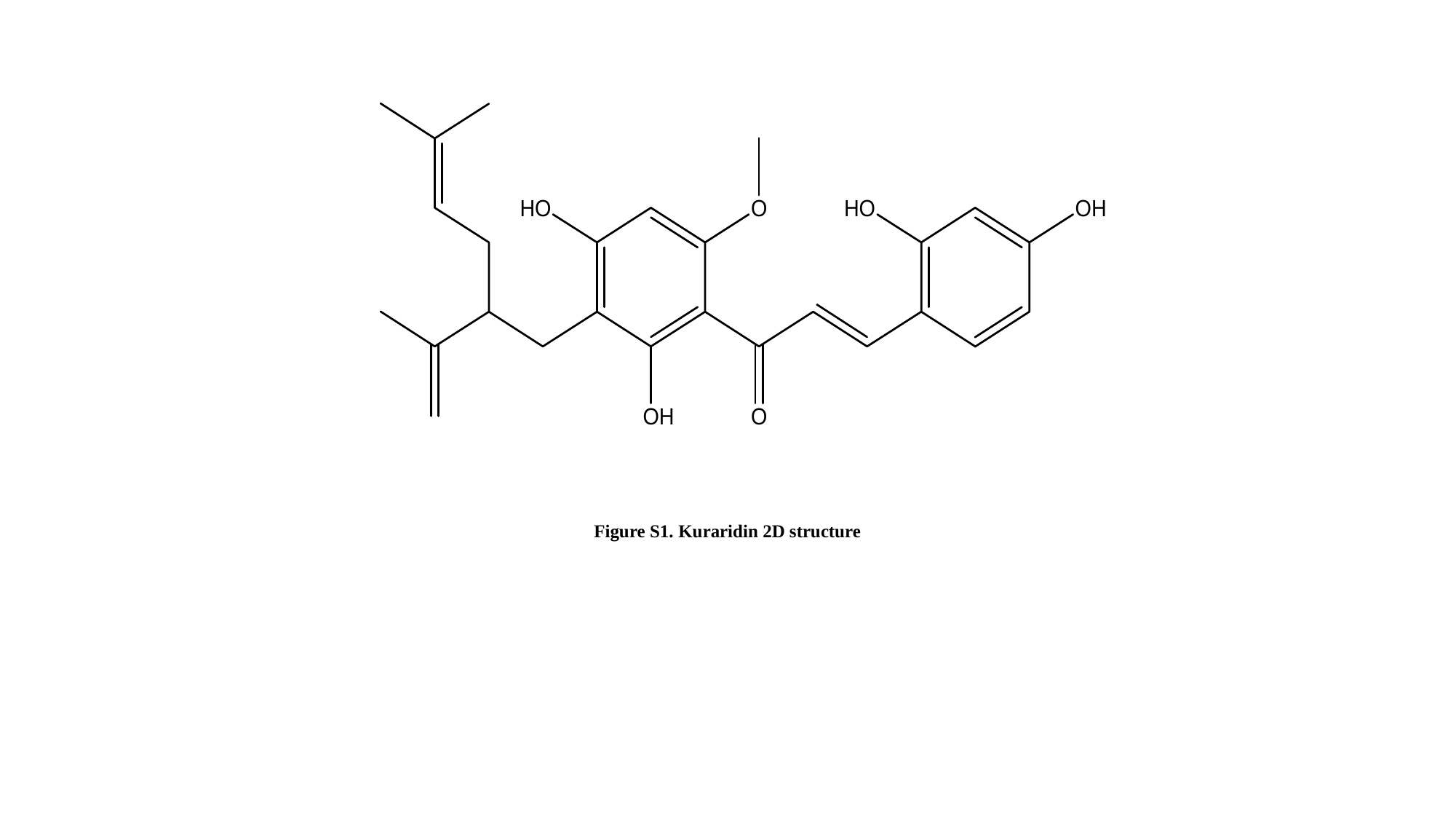

Figure S1. Kuraridin 2D structure

## Slide 2
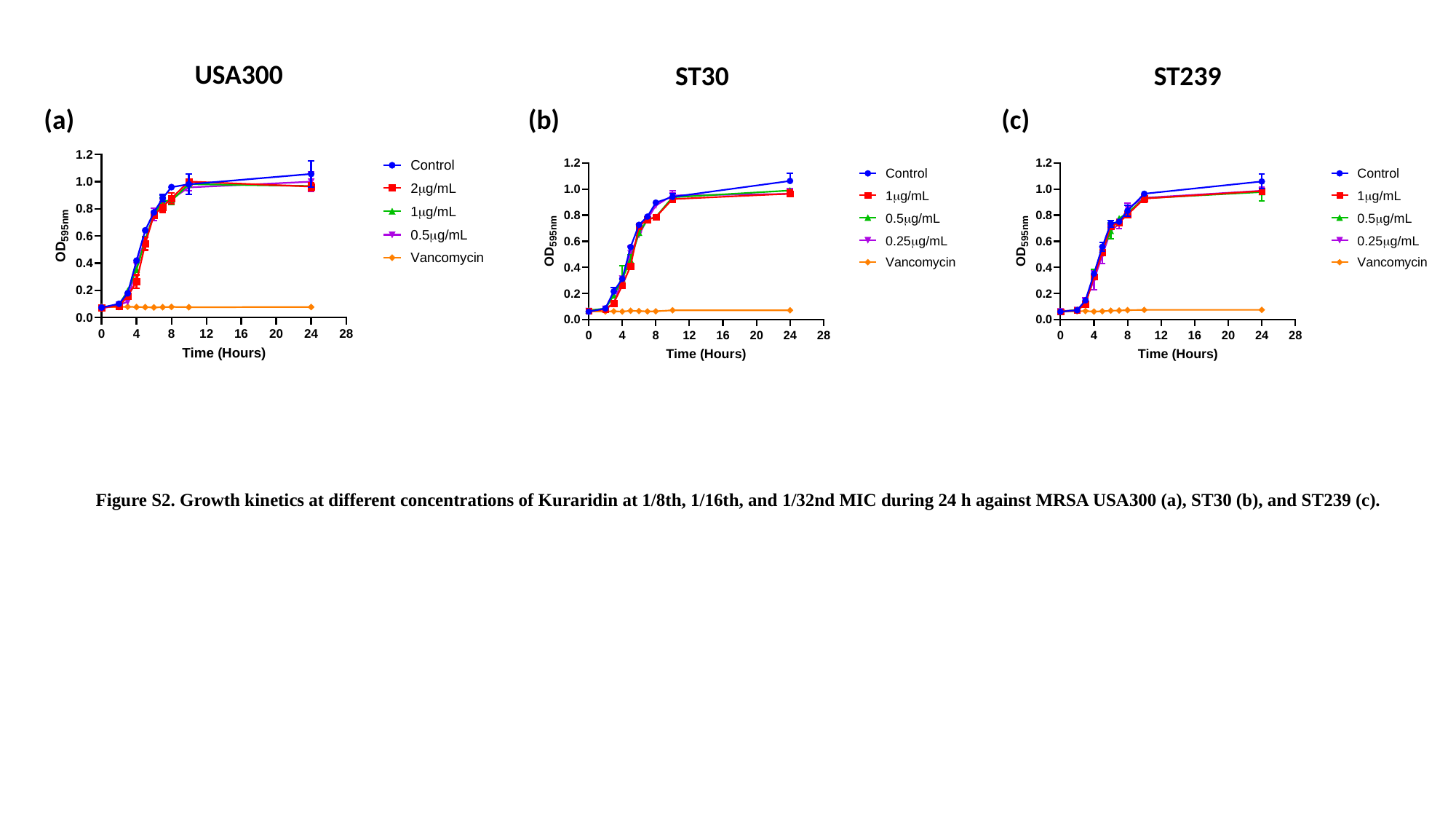

USA300
ST30
ST239
(a)
(b)
(c)
Figure S2. Growth kinetics at different concentrations of Kuraridin at 1/8th, 1/16th, and 1/32nd MIC during 24 h against MRSA USA300 (a), ST30 (b), and ST239 (c).

## Slide 3
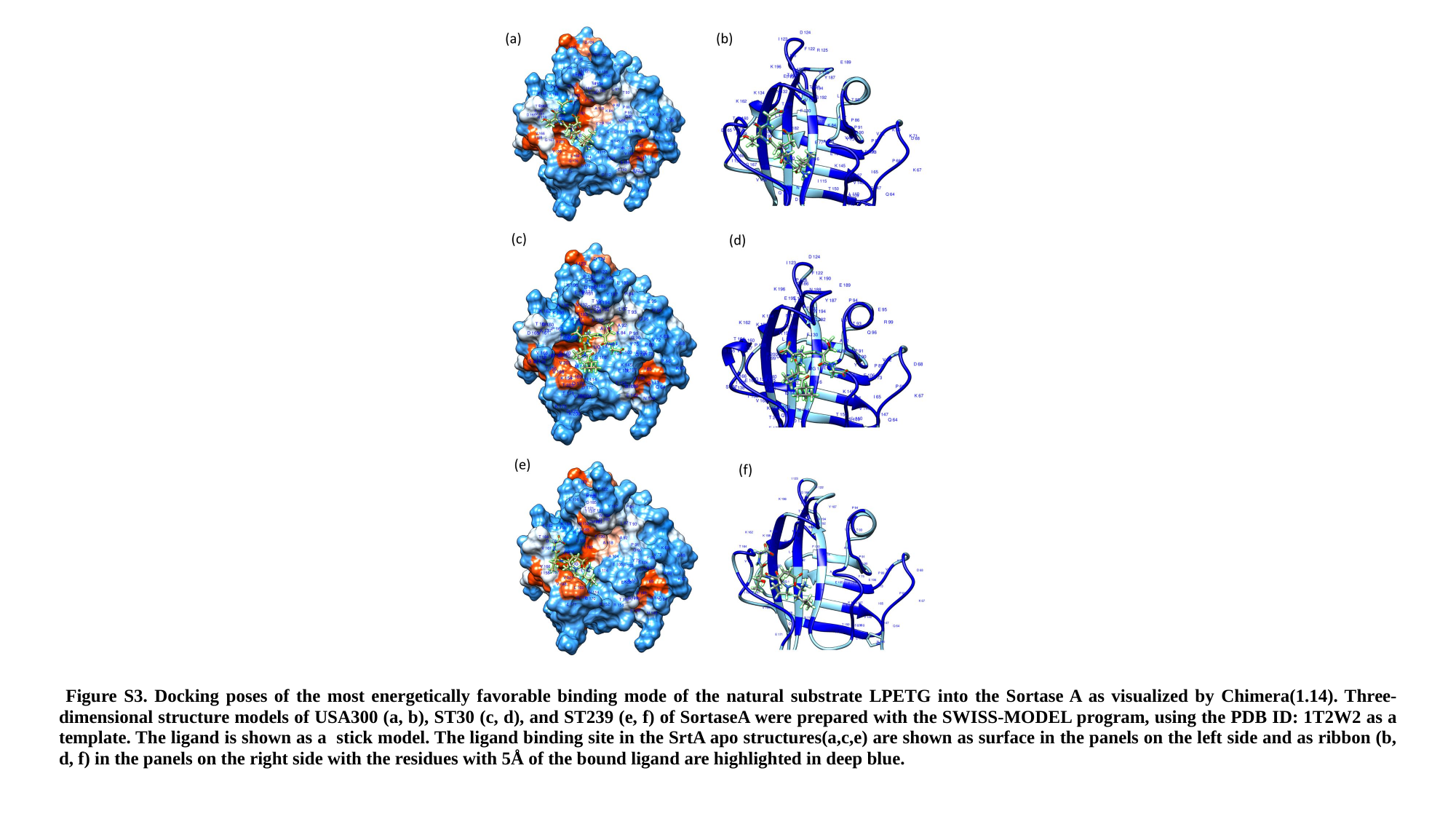

Figure S3. Docking poses of the most energetically favorable binding mode of the natural substrate LPETG into the Sortase A as visualized by Chimera(1.14). Three-dimensional structure models of USA300 (a, b), ST30 (c, d), and ST239 (e, f) of SortaseA were prepared with the SWISS-MODEL program, using the PDB ID: 1T2W2 as a template. The ligand is shown as a stick model. The ligand binding site in the SrtA apo structures(a,c,e) are shown as surface in the panels on the left side and as ribbon (b, d, f) in the panels on the right side with the residues with 5Å of the bound ligand are highlighted in deep blue.

## Slide 4
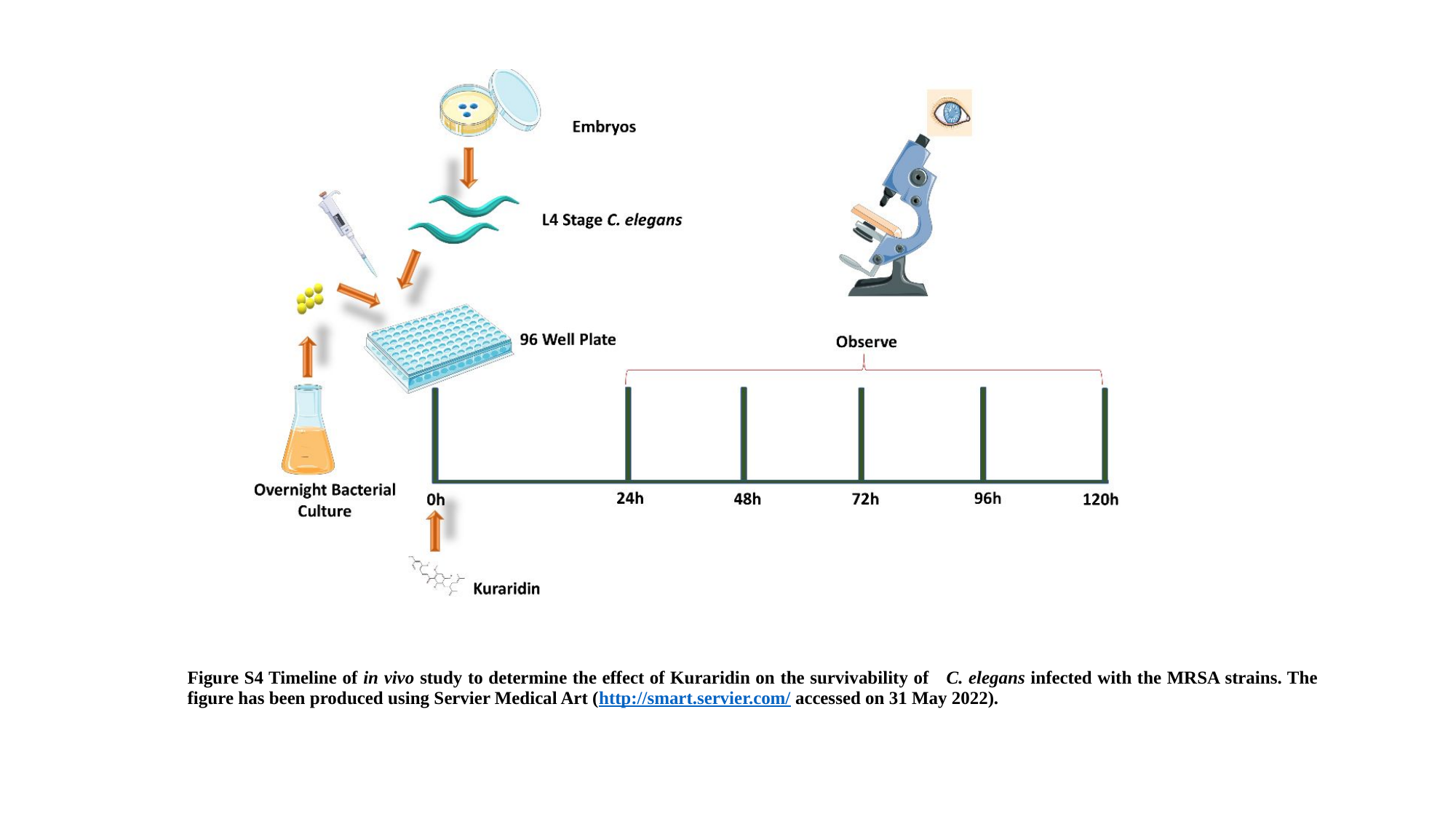

Figure S4 Timeline of in vivo study to determine the effect of Kuraridin on the survivability of C. elegans infected with the MRSA strains. The figure has been produced using Servier Medical Art (http://smart.servier.com/ accessed on 31 May 2022).

## Slide 5
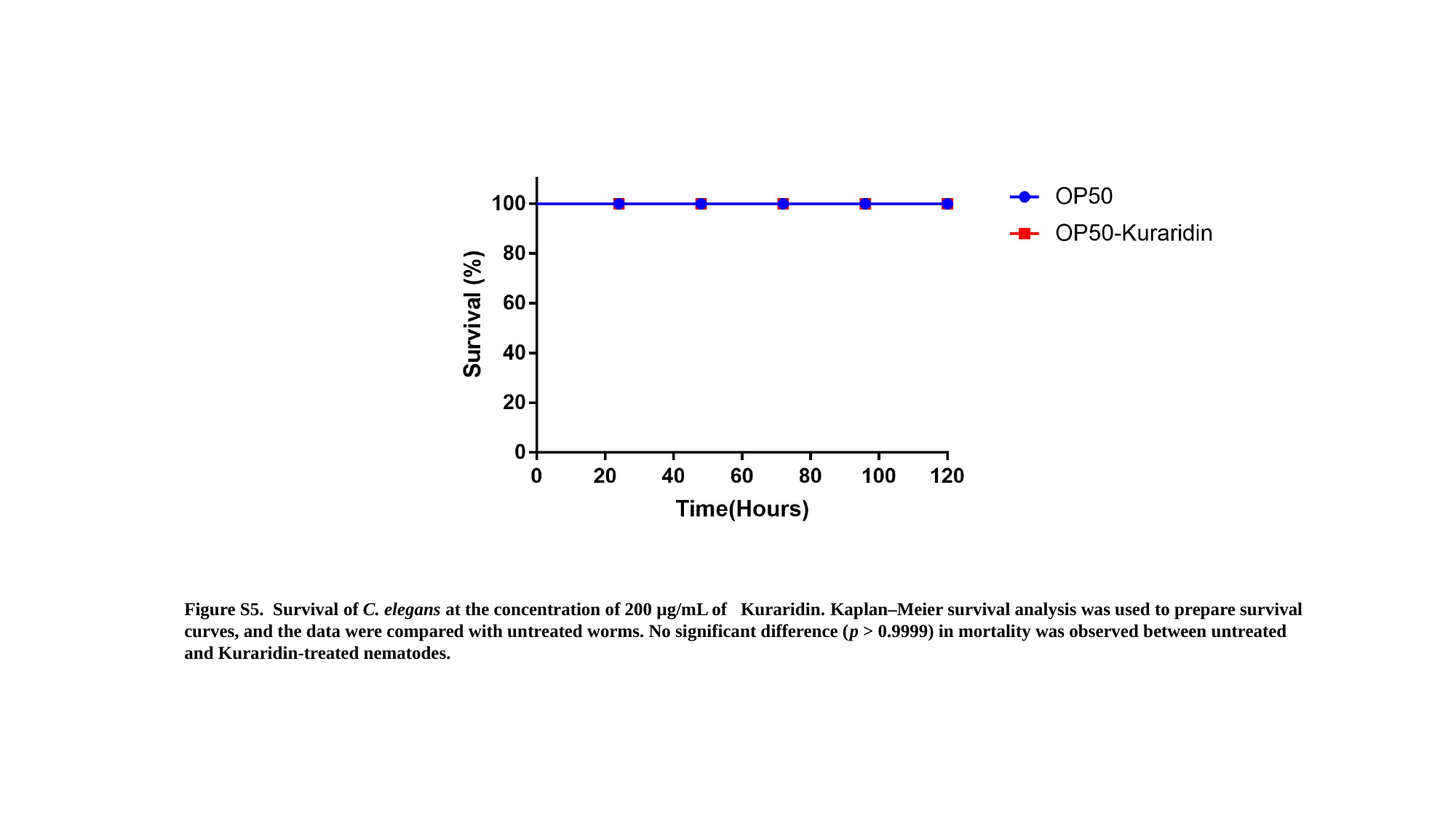

Figure S5. Survival of C. elegans at the concentration of 200 µg/mL of Kuraridin. Kaplan–Meier survival analysis was used to prepare survival curves, and the data were compared with untreated worms. No significant difference (p > 0.9999) in mortality was observed between untreated and Kuraridin-treated nematodes.

## Slide 6
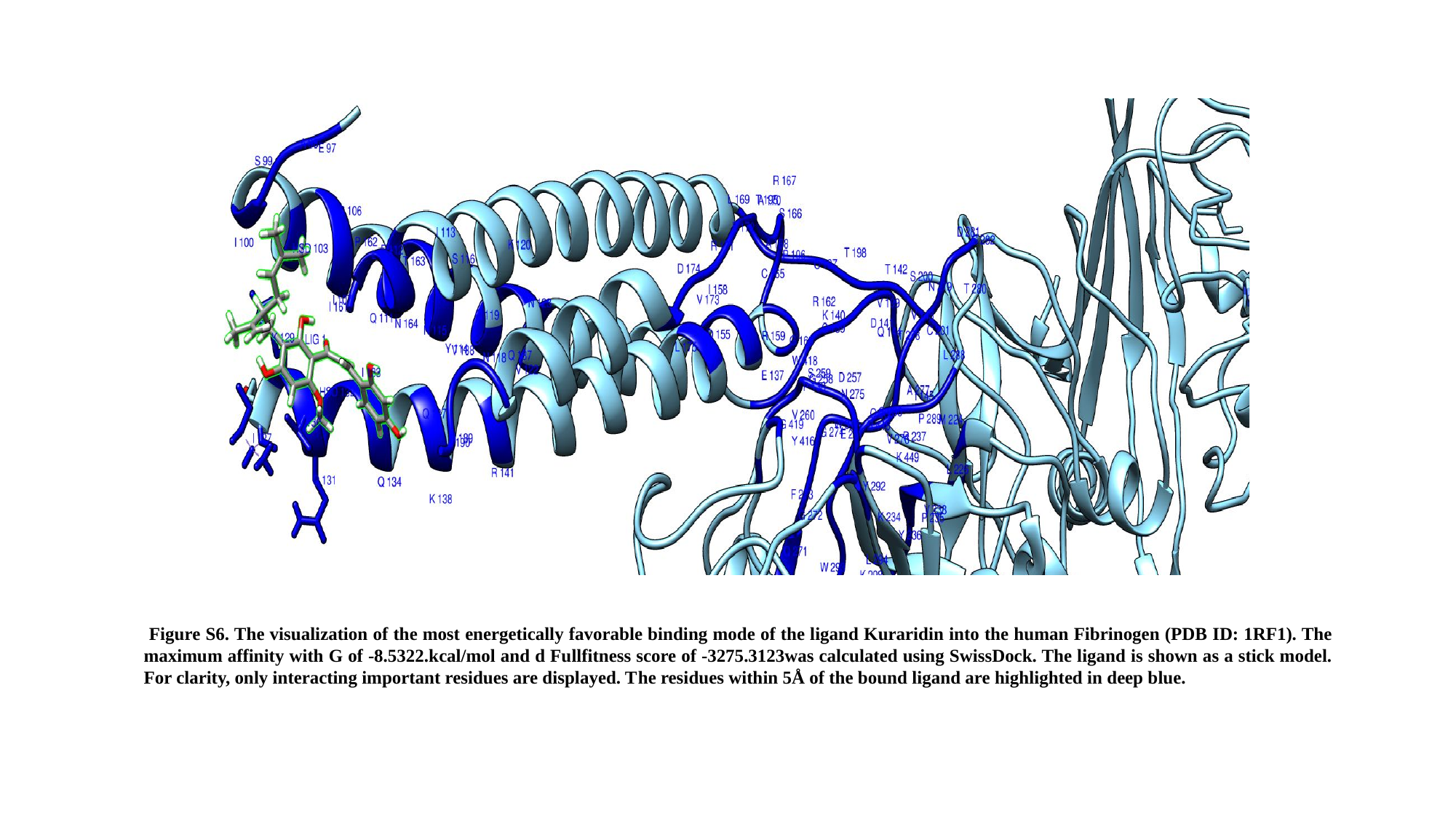

Figure S6. The visualization of the most energetically favorable binding mode of the ligand Kuraridin into the human Fibrinogen (PDB ID: 1RF1). The maximum affinity with G of -8.5322.kcal/mol and d Fullfitness score of -3275.3123was calculated using SwissDock. The ligand is shown as a stick model. For clarity, only interacting important residues are displayed. The residues within 5Å of the bound ligand are highlighted in deep blue.
